# The Advantage of PET and CT Integration in Examination of Lung Tumors

**DOI:** 10.1155/2007/17131

**Published:** 2007-11-12

**Authors:** Guangming Lu, Zhongqiu Wang, Hong Zhu, Linfeng Chang, Yingxin Chen, Jiang Wu, Yane Zhao

**Affiliations:** Department of Medical Imaging, Jinling Hospital, Clinical School of Medical College, Nanjing University, Nanjing 210002, China

## Abstract

*Purpose.* To evaluate the diagnosis value of integrated positron emission tomography and computed tomography (PET/CT) with lung masses, this study emphasized the correlation between tumor size and maximum standardized uptake value (SUVmax) in selected regions of interest (ROI) of lung masses. *Material and Methods.* A retrospective analysis was performed on 85 patients with solid pulmonary lesions, all verified by pathology. The morphology, edge (speculated margins and lobule), size, density of pulmonary masses, and on-chest CT images were reviewed. The SUVmax in ROI of pulmonary masses was calculated. *Results.* Among the 85 patients with lung masses, 59 patients presented with pulmonary malignant neoplasm and 26 patients with benign lesions. The sensitivity, specificity, and accuracy were 89.8%, 61.5%, 81.2%, respectively, for PET measurement only, 88.1%, 65.4%, 81.2% for CT only, and 96.6%, 80.8%, 91.8% for PET/CT. The size of pulmonary malignant neoplasm in the 59 patients was apparently correlated with the ROI's SUVmax (r=0.617, P<.001). However, the size of pulmonary benign mass in the 26 patients was not correlated with the SUVmax. *Conclusion.* PET/CT is of greater value in characterization of lung masses than PET and CT performed separately. The examination of lung tumor can be further specified by the correlation between the size of pulmonary malignant neoplasm and the ROI's SUVmax.

## 1. THE ADVANTAGE OF PET AND CT INTEGRATION IN EXAMINATION OF LUNG TUMORS

In recent years, the incidence and mortality of lung cancer are always ranked as the highest among all neoplasms. Mass is the principal manifestation of lung cancer, whose diagnosis is of vital clinical significance [1–5]. Early and accurate diagnosis of lung cancer is critical to its therapy. PET/CT combines the merits of both functional and anatomical imaging techniques and has been widely used in clinical examination, with a hope to make the diagnosis of neoplasm as early as possible. The current study evaluated the diagnosis value of PET/CT.

## 2. MATERIAL AND METHODS

### 2.1. Imaging acquisition

Fluorine-18-labeled fluorodeoxyglucose (18F-FDG) was produced by EBCO cyclotron facility. Radiochemical purity (>95%) of 18F-FDG was verified by analytical HPLC. All patients fasted for at least 6 hours before PET/CT examination. After ensuring a normal peripheral blood glucose level, patients received an intravenous injection of 0.2 mCi/kg of 18F-FDG, and then rested for approximately 50–60 minutes before undergoing a PET/CT scan. Image acquisition was performed using an integrated PET/CT device (Siemens Biograph Sensation 16). CT was performed from the head to the pelvic floor using a standardized protocol (120 KV, 80 mA with a slice thickness of 5 mm). PET images in early display were acquired using 3D mode for the same scanning range as CT. The acquisition time for PET was 3 minutes per bed position and 5-6 continuous positions were scanned. Delayed images of chest were acquired at 3 hours after injection of 18F-FDG. The acquisition parameters of the two PET scans are the same. PET images datasets were reconstructed iteratively using an ordered subset expectation maximization algorithm and corrected with measured attenuation correction. The SUVmax of the selected ROI in lesions was calculated. CT, PET, and PET/CT infusion images of axial, sagittal, and coronal images were obtained through a postprocessing procedure.

### 2.2. Patient data

85 patients (54 males, 31 females; age range: 36–87 years; mean age: 58 years) with lung masses were enrolled in this study. Each patient received the early 18-FDG scan described above. 70 patients underwent a second delayed scan because either their pulmonary masses could not be determined or they had suspected pulmonary malignancies. Other 15 patients did not undergo delayed 18F-FDG scan because they already had a definite diagnosis based on CT and/or early PET scan.

### 2.3. Data analysis and processing

#### 2.3.1. Semiautomatic quantification of ROI

ROI was drawn on the slice that showed clearly radioactivity aggregation. For an early scan, the SUVmax over 2.5 was regarded as positive; and for a delayed scan, the SUVmax over 2.5 or with10% increase compared to the early scan, was recognized as positive. CT images were mainly employed to examine the morphology, edge (i.e., speculated margins and lobulation), size, and density of pulmonary lesions. Metastasis derived from PET or CT was comprehensively analyzed with other clinical profiles. If a lung mass was irregular in its shape and/or its edge was poorly defined such as being spiculated, having radiating corona, umbilicated, or lobulated without benign signs of lung masses (e.g., having characteristic calcification or fat), it was regarded as malignant neoplasm. The images were interpreted by two experienced radiologists who had obtained a position higher than the rank of attending physicians. Diagnosis was determined only when a consensus was achieved. If no consensus was achieved, it would be subject to further review by the whole department. The diagnostic sensitivity, specificity, and accuracy of PET only, CT only, and PET/CT were analyzed. The correlations between tumor sizes and ROI' SUVmax were quantitatively compared.

### 2.4. Statistics

The correlations between tumor sizes and metabolism of the lesions were performed using an SPSS software (version 11.5). Pearson correlation was calculated with P<.05 or P<.001, considered as a standard of significance level or a very significant difference, respectively.

## 3. RESULTS

There were 85 patients with solid pulmonary lesions: 59 cases of malignant neoplasms, 26 cases of benign masses. Among the malignant neoplasm cases, there were 19 squamocellular carcinomas, 25 adenocarcinomas, 3 alveolar cell carcinomas, 4 small-cell nondifferentiated adenocarcinomas, 1 eosinophilic cell carcinoid, 1 adenospuamous carcinoma, 2 dual-origin carcinomas (one right and left upper lung cavernous squamocellular carcinoma and one left upper lung adenocarcinoma with right lower lung mixed carcinoma), and 4 metastases. Among the benign mass cases, there were 9 lung tuberculoses, 7 inflammatory granulomatosis, 3 chronic inflammations, 2 acute inflammations, 1 round ateletasia, 1 fungus, and 3 other benign tumors. The diagnostic values of PET only, CT only, and PET/CT for these lung masses were shown in Table 1 and Figures 3(a)–5(a). There were 10 false positive cases and 6 false negative cases (5 neoplasms with a diameter lower than 1 cm and one highly differentiated carcinoid) for PET-only imaging. 9 false positive cases and 7 false negative cases would have been found if CT-only scan was employed. The numbers of both false positive cases (5 cases) and false negative cases (2 cases) for integrated PET/CT were smaller than those for PET alone or CT alone (see Table 1 and Figures 3–5).

**Figure 1:**
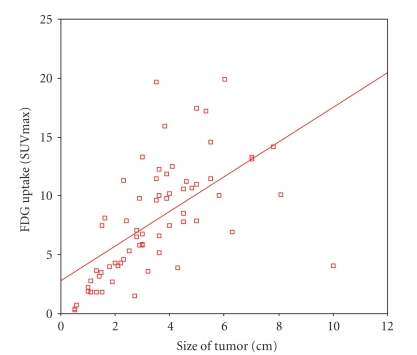
The correlation between FDG uptake and the sizes of 59 malignant tumors.

**Figure 2:**
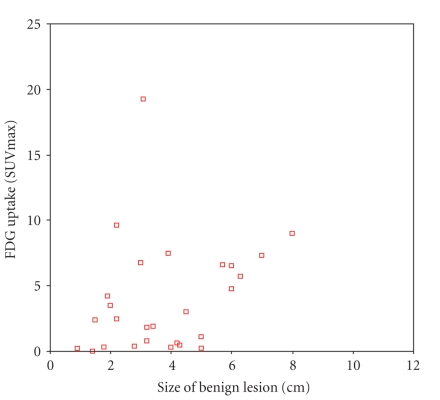
The correlation between FDG uptake and the sizes of 26 benign masses.

**Figure 3:**
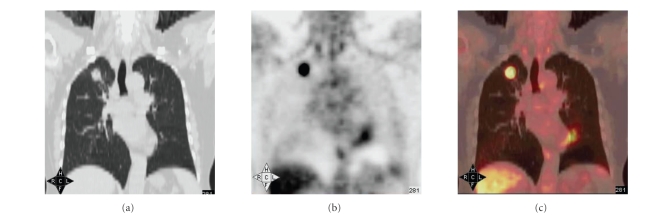
(a), (b), and (c) were from the same patient. The pathological diagnosis is right upper pulmonary squamous carcinoma. There were typical manifestations on CT, PET, and integrated PET/CT. The mass on CT is 2.4 cm×2.5 cm. The SUVmax of early PET imaging is 7.8.

**Figure 4:**
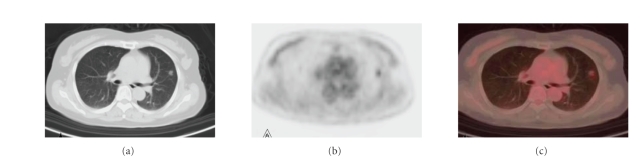
(a), (b), and (c) were from the same patient. The pathological diagnosis is left upper lung adenocarcinoma. On CT, a small module (0.9 cm×1.0 cm) with lobulation and speculated margin was seen in the left upper lung (a). No typical manifestation was seen on PET. The SUVmax of early PET imaging was 1.9 (b). Integrated PET/CT suggested suspected lung carcinoma (c).

**Figure 5:**
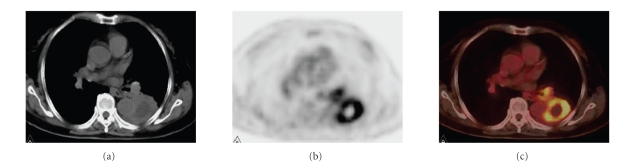
(a), (b), and (c) were from the same patient. The pathology is left lung inflammation. A big mass (5.3 cm×5.7 cm) with lobulation and spurring was seen on CT (a). Malignant tumor was suspected on CT. False positivity was seen on PET; its early imaging SUVmax is 6.6 (b). The integrated PET/CT also suggested possible lung carcinoma (c).

Figures 1 and 2 illustrate the correlations between 18F-FDG uptake and the tumor sizes. The results from a statistical analysis showed that while the sizes of pulmonary malignant tumors were significantly correlated with the ROI' SUVmax (r=0.617, P<.001), there was no significant correlation between the ROI' SUVmax and the sizes of masses in benign lesions measured on PET/CT.

## 4. DISCUSSION

Lung masses might be classified when they are larger than 3 cm and less than 3 cm in diameter. Among the solitary pulmonary nodules with a diameter less than 3 cm, there were 33% malignant nodules, 54% inflammatory granulomatoses, 6% hamartomas, 5% isolated metastases, and 2% bronchial adenomas [3–7]. Most lung masses with a diameter larger than 3 cm were malignant [4]. To resolve differential diagnoses of lung masses based on the different types of scans still remains a challenge to radiologists.

### 4.1. The diagnostic value of PET alone for lung mass

The extent of 18F-FDG uptake can be a good reference to the property of a certain mass. The absorbances of FDG in malignant neoplasms were significantly higher than those of benign tumors. Most radiologists employed the semiautomatic quantification of SUVmax, with 2.5 as a threshold value [1, 2, 8, 9]. Tumors with SUVmax >2.5 were classified as malignant lesions. End-stage pulmonary carcinoma can be accompanied with high metabolism metastasis to pulmonary hilar lymph nodes, mediastinal lymph nodes, and other organs [9, 10]. By applying this standard, the diagnostic sensitivity, specificity, and accuracy are about 89.8%, 61.5%, and 81.2%, respectively. Dewan et al. [11] reported that the diagnostic sensitivity, specificity, and accuracy of PET for lung nodules were 95%, 87%, and 92%, respectively. In 1474 cases with solitary pulmonary nodules, the 18F-FDG PET had a diagnostic sensitivity of 96.8% and a specificity of 77.8% [12]. While discrepancy exists between our work and other reports, false positivity and false negativity exist in all groups. Tuberculosis, inflammatory pseudotumor, aspergillosis, and granulomatosis can also have an uptake of FDG and lead to false positivity. In our cases, there were 10 false positive cases, which had a lower positive predictive value of 85.2% for PET. Among the 6 false negativities, 5 were small lung cancer (diameter <10 mm), which implies that the threshold of SUVmax 2.5 needs to be modified for the diagnosis of small lung cancer with PET. In addition, dual-time-point 18F-FDG PET is necessary in order to improve the accuracy of diagnosis.

### 4.2. Diagnosis of lung mass with CT

CT scan was used to analyze the characteristics of lesions involving the location, morphology, edge (i.e., speculated margins and lobule), size, density, and enhancement manifestations after injecting contrast agent. Small lung nodules require a thin-slice CT scan protocol and/or dynamic enhancement. Although spiral CT could afford more detailed information such as intranodular calcification and blood supply of the mass, it still lacks specificity for certain lung nodules. Our results showed that the sensitivity, specificity, and accuracy of CT in the diagnosis are 88.1%, 65.4%, and 81.2%, respectively. Yi et al. [13] reported that the diagnostic sensitivity, specificity, and accuracy of lung malignant neoplasm with dynamic enhanced spiral CT are 81%, 93%, and 85%, respectively. Though its diagnostic value improved somewhat, there is still some difficulty in evaluating their cases. There were 9 false positive cases and 7 false negative cases in our CT series. The reason for this difference might be that the findings based on pathologies could have a similar CT manifestation while the same pathology might have different images. Therefore, to improve the diagnostic accuracy, other diagnostic procedures need to be integrated with CT measurements.

### 4.3. The correlation of lung mass size and its metabolism

This research showed that there was a positive correlation between the size of malignant tumor and PET/CT SUVmax (r=0.617, P<.001). In Figure 1, there is a linear correlation between the malignant tumor size and SUVmax. Not only tumor size but also the focal metabolism should be taken into account in the diagnosis of malignant tumors with PET/CT. Especially for those with an SUVmax <2.5, lung carcinoma could not be excluded. False negativity might arise from the following reasons [6, 7, 9, 14 –17].

(1) Some types of tumors, for example, bronchial alveolar cell carcinoma, carcinoid, and well-differentiated adenocarcinoma, might have a reduced metabolism, and a false negativity.

(2) Tumors smaller in diameter (<10 mm) have a low SUVmax, and they might produce partial volume effect.

(3) There are a large number of fibers inside the tumors and a low quantity of tumor cells.

(4) The patients had high blood glucose level.

Most false negativities in our series were at small foci. Great caution should be taken for the diagnostic small nodules with a diameter <10 mm since such small malignancies might have an SUVmax <2.5. Different criteria are needed to determine malignancy in nodules less than 10 mm in diameter. As shown in Figure 2, there is no correlation between the size of benign lesions and SUVmax of PET/CT. This may reflect the complexity of tumor metabolism, suggesting that the masses are not proportional to SUVmax of PET/CT. If a focus has an SUVmax >2.5, but the size of the mass is not in accordance with the SUVmax, caution should be taken to avoid false positivity. Bunyaviroch et al. [6, 7, 9, 10, 17] reported that false positivity in imaging might arise in tuberculosis, sarcoidosis, histoplasmosis, aspergillosis, and pleural mesothelioma.

### 4.4. Diagnostic value of integrated PET/CT for lung tumor

The diagnostic procedure of integrated PET/CT for lung masses was as follows.

(1) The metabolism of FDG should follow the standard for malignant tumors from the view of PET.

(2) Lung tumors should follow the standard of CT concerning the density, morphology, edge, and enhancement.

When either of the standards was met, lung tumor could be diagnosed. When only one of the requirements was achieved, caution should be taken. Further, inspection should pursue. When neither of the requirements was reached, lung tumor could be excluded. Yi et al. [13, 16] reported that the diagnostic sensitivity, specificity, and accuracy of integrated PET/CT for lung malignancy were 96%, 88%, and 93%, respectively, but 96.6%, 80.8%, and 91.8% in our series. Winer-Muram et al. [16, 18] reported that as compared with CT, PET/CT provided additional information, including more accurate location, differentiation of pathological and physiological uptakes, pickup of foci omitted by CT. The results from the current study showed that integrated PET/CT had higher sensitivity and specificity than CT or PET when performed separately, indicating that PET/CT may play a more important role in lung tumor diagnosis. Given the false positivity and false negativity detected, PET/CT may not be ideally specific for lung tumor [13, 15, 16, 18]. We ought to combine the information of focal metabolism, morphology, volume, and density in order to avoid false positivity and false negativity. To meet such need, the usage of different tracers, needle biopsy, or follow-up should be pursued to ensure accurate diagnosis.

## 5. CONCLUSION

The integration of PET and CT is of greater value for the diagnosis of lung masses than other methods using PET or CT alone. Our results showed that the size of pulmonary malignant neoplasms was correlated with ROI' SUVmax of PET/CT positively, but the size of pulmonary benign lesion was not correlated with the SUVmax. These findings indicate that PET/CT may enhance the sensitivity, specificity, and accuracy of diagnosis on lung tumors.

## Figures and Tables

**Table 1:** The diagnostic value of PET only, CT only, and integrated PET/CT on 85 patients with lung masses.

Methods	Sensitivity (%)	Specificity (%)	Positive predictive value (%)	Negative predictive value (%)	Accuracy (%)
PET only	89.8 (53/59)	61.5 (16/26)	84.1 (53/63)	72.7 (16/22)	81.2 (69/85)
CT only	88.1 (52/59)	65.4 (17/26)	85.2 (52/61)	70.8 (17/24)	81.2 (69/85)
Integrated PET/CT	96.6 (57/59)	80.8 (21/26)	91.9 (57/62)	91.3 (21/23)	91.8 (78/85)

See Figures 3(a)–5(c) for further demonstration of our cases.
